# Plasma protein expression differs between colorectal cancer patients depending on primary tumor location

**DOI:** 10.1002/cam4.3178

**Published:** 2020-05-26

**Authors:** Matilda Holm, Sakari Joenväärä, Mayank Saraswat, Tiialotta Tohmola, Ari Ristimäki, Risto Renkonen, Caj Haglund

**Affiliations:** ^1^ Department of Surgery Faculty of Medicine University of Helsinki and Helsinki University Hospital University of Helsinki Helsinki Finland; ^2^ Department of Pathology Faculty of Medicine University of Helsinki and Helsinki University Hospital University of Helsinki Helsinki Finland; ^3^ Translational Cancer Medicine Research Program Faculty of Medicine University of Helsinki Helsinki Finland; ^4^ Applied Tumor Genomics Research Program Faculty of Medicine University of Helsinki Helsinki Finland; ^5^ Transplantation Laboratory Haartman Institute University of Helsinki Helsinki Finland; ^6^ HUSLAB Helsinki University Hospital Helsinki Finland; ^7^ Department of Laboratory Medicine and Pathology Mayo Clinic Rochester Minnesota USA; ^8^ Department of Biosciences Faculty of Biological and Environmental Sciences University of Helsinki Helsinki Finland

**Keywords:** colorectal cancer, left colon, mass spectrometry, plasma proteomics, rectum, right colon

## Abstract

Colorectal cancer (CRC) includes tumors in the right colon, left colon, and rectum, although they differ significantly from each other in aspects such as prognosis and treatment. Few previous mass spectrometry‐based studies have analyzed differences in protein expression depending on the tumor location. In this study, we have used mass spectrometry‐based proteomics to analyze plasma samples from 83 CRC patients to study if differences in plasma protein expression can be seen depending on primary tumor location (right colon, left colon, or rectum). Differences were studied between the groups both regardless of and according to tumor stage (II or III). Large differences in plasma protein expression were seen, and we found that plasma samples from patients with rectal cancer separated from samples from patients with colon cancer when analyzed by principal component analysis and hierarchical clustering. Samples from patients with cancer in the right and left colon also tended to separate from each other. Pathway analysis discovered canonical pathways involved in lipid metabolism and inflammation to be enriched. This study will help to further define CRC as distinct entities depending on tumor location, as shown by the widespread differences in plasma protein profile and dysregulated pathways.

## Graphical abstract

Colorectal cancer (CRC) includes tumors in the right colon, left colon, and rectum, although they differ significantly from each other in aspects such as prognosis and treatment. In this study, we have used mass spectrometry‐based proteomics to analyze plasma samples from 83 CRC patients. We subsequently show that plasma protein expression differs significantly between patients with cancer in the colon and rectum.

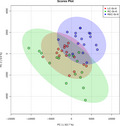

## 1 BACKGROUND

Colorectal cancer (CRC) is the third most common cancer worldwide and the second most common cause of cancer death, with over 1.8 million new cases and closer to 900 000 deaths estimated to have occurred in 2018. CRC therefore accounts for 10% of the global cancer burden, and the burden is expected to increase by 60% to more than 2.2 million new cases and 1.1 million deaths by 2030.^1^, ^2^ CRC includes cancer in the colon, which is further divided into the right and left colon, and the rectum. Cancer in the right colon differs from cancer in the left colon, and rectal cancer differs from colon cancer in multiple ways.^3^ The boundary between the right and left colon, which is defined by embryological origin, is the distal transverse colon. Seeing as this is difficult to use in retrospective analyses, most studies use the splenic flexure as the boundary between cancer in the right colon and left colon, with tumors arising proximal to the splenic flexure being classified as right‐sided and tumors arising distal to the splenic flexure being classified as left‐sided.^4^, ^5^

Cancer in the right and left colon differ in multiple ways, with some proposing that they should be regarded as different entities.^6^, ^7^ Right‐sided colon cancer is more often seen in older people and women, and patients tend to present with more advanced tumor stage and more subtle symptoms.^8^, ^9^ Several studies have also shown that right‐sided colon cancer has a worse prognosis than left‐sided colon cancer, although the reason for this is unclear.^10^, ^11^, ^12^ Rectal cancer has a greater risk of local recurrence as resection is harder due to anatomical constraints. It also has a greater risk of metastasizing to the lungs than colon cancer.^13^, ^14^ However, the 5‐year survival rates for colon and rectal cancer are similar, with rectal cancer actually having a slightly higher 5‐year survival rate (66.6%) than colon cancer (63.6%).^15^ Differences between rectal and colon cancer are also apparent at the molecular level. Rectal tumors more often display mutations in genes such as tumor protein p53 (*TP53*), while colon tumors more often have mutations in genes including B‐Raf proto‐oncogene (*BRAF*).^14^, ^16^ The consensus molecular subtypes (CMS) are a recent classification of CRC into four subtypes with distinguishing features considered the most robust classification system currently available for CRC. CMS1 tumors, which are characterized by microsatellite instability (MSI), the CpG island methylator phenotype (CIMP), and hypermutation, were frequently right‐sided. CMS2 tumors, which display higher chromosomal instability than CMS1 and upregulation of *WNT* and *MYC* downstream targets, were mainly left‐sided.^17^

Few previous mass spectrometry‐based studies have analyzed differences in protein expression depending on tumor location.^18^ One study used mass spectrometry to analyze tissue samples from patients with cancer in the right and left colon and discovered that the expression of proteins involved in cellular energy metabolism, protein folding, and oxidative stress varied between samples from the two locations.^19^ Another study revealed distinct protein expression between right‐ and left‐sided colon cancer and identified several proteins that could be of use in predicting relapse in right‐ or left‐sided colon cancer.^20^ In this study, we have used the Ultra Performance Liquid Chromatography‐Ultra Definition Mass Spectrometry (UPLC‐UDMS^E^)‐based proteomics to analyze plasma samples from 83 CRC patients who were divided into groups based on if they had tumors in the right colon, left colon, or rectum. The samples were compared between primary tumor locations, both regardless of and according to tumor stage (II or III), and plasma protein expression was analyzed. The aim of this study was to investigate whether differences were seen in plasma protein expression between patients with tumors in the right colon, left colon, or rectum. While a previous study has investigated how plasma protein expression changes during stage II and III CRC,^21^ both depending on and regardless of tumor location, as far as we are aware, this study is the first to show that plasma protein profiles differ significantly between CRC patients depending on tumor location.

## 2 MATERIALS AND METHODS

### 2.1 Patient samples

This study used preoperative plasma samples from a total of 83 CRC patients with stage II or III cancer in the right colon, left colon, or rectum. Cancer staging was performed according to the TNM staging system. Stage II cancer was defined as cases with a pT3‐4 primary tumor but no regional lymph node or distant metastasis, while stage III cancer was defined as cases with regional lymph node metastasis but no distant metastasis. The patients underwent surgical resection with curative intent between 2000 and 2007. The patients included in this study were all newly diagnosed with stage II or III cancer. Patients with conditions including other types of cancer, a previous history of hereditary nonpolyposis colorectal cancer (HNPCC), familial adenomatous polyposis (FAP), ulcerative colitis, Crohn's disease, or mucinous tumors were also excluded from this study. Plasma samples were stored at −80°C until processed as described below. The clinical data were obtained from patient records, the survival data from the Population Register Centre of Finland, and the cause of death for all the deceased from Statistics Finland. Written informed consent was obtained from all patients prior to collecting samples. This study was approved by the local Surgical Ethics Committee and carried out in accordance with the Declaration of Helsinki.

### 2.2 Sample processing and digestion

The plasma samples were processed as described previously^21^, ^22^ and as follows. All samples were thawed and top 12 protein depletion was performed with TOP12 protein depletion spin columns (85 165, Pierce) according to the manufacturer's instructions. After the total protein concentration was determined, plasma equivalent to 100 µg protein was aliquoted and dried. The samples were dissolved in 35 µL Tris buffer (50 mmol/L, pH 7.8, T1503, Sigma) containing 6M urea (51 459, Fluka), after which 1.8 µL of dithiothreitol (DTT, 200 mmol/L, V315B, Promega) was added. After the samples were shaken for 1 hour at room temperature, 7 µL of iodoacetamide (200 mmol/L, 57 670, Fluka) was added to each sample and they were returned to the shaker for another hour. After this, 7 µL of DTT (200 mmol/L) was added to each sample and the samples were again put on the shaker for 1 hour. The samples were subsequently diluted with 270 µL mQ water per sample. Trypsin (V5280, Promega) was added at a ratio of 1:50 trypsin to protein. Digestion was carried out at 37°C overnight, and the next day C18 spin columns (89 870, Pierce) were used to clean 30 µg of tryptic peptides per sample. These peptides were dissolved in 86 µL of 0.1% formic acid that contained 12.5 fmol/µL of Hi3 spike‐in standard peptides (186 006 012, Waters) for quantification.

### 2.3 Ultra performance liquid chromatography‐ultra definition mass spectrometry and quantification UPLC‐UDMS^E^

UPLC was performed as described previously^21^, ^22^ and as follows. Four microliter of each sample (the equivalent to ~1.4 µg total protein) was injected to a nanoACQUITY UPLC system (Waters Corporation,) and TRIZAIC nano‐Tile 85 μm × 100 mm HSS‐T3u wTRAP was used as a separation device. As mentioned in Holm et al^21^: “Samples were loaded, trapped, and washed for two minutes with 8.0 µL 1% B and the analytical gradient used was as follows: 0‐1 minutes 1% B, at 2 minutes 5% B, at 65 minutes 30% B, at 78 minutes 50% B, at 80 minutes 85% B, at 83 minutes 85% B, at 84 minutes 1% B, and at 90 minutes 1% B with 450 nL/min. Buffer A was 0.1% formic acid in water while buffer B was 0.1% formic acid in acetonitrile. Data were acquired in data‐independent acquisition fashion using UDMSE mode with a Synapt G2‐S HDMS (Waters Corporation).”

UDMS^E^ data acquisition mode was used in this study to optimize the collision energies, which has previously been described by Distler et al.^23^ Briefly, in classical HDMS^E^, one fixed collision energy is applied to each individual ion mobility separation cycle, which results in under‐ or over‐fragmentation of precursor ions. Distler et al^23^ devised a strategy for using linear regression to specify drift‐time specific collision energies for every drift‐time bin of the IMS cycle, resulting in optimized energies for all precursors. Calibration was performed with sodium iodide clusters over a mass range of 50‐2500 m/z. A solution of 2 µg/µL sodium iodide in 50/50 2‐propanol/water was infused into the mass spectrometer. 10% of the samples were run in triplicate and the median coefficient of variation (%CV) of the dataset was 4.36%.

### 2.4 Data analysis

Data analysis and label‐free quantification were performed as described previously.^21^, ^22^, ^24^ The raw files were imported to Progenesis QI for proteomics, version 4.1 (Nonlinear Dynamics).^25^ Post‐acquisition mass correction was performed when the raw data were imported, using a lock mass ion of M + H+ 556.2771 m/z, with leucine enkephalin (C25H37O7, 1 ng/µL in 50:50 acetonitrile:water + 0,1% formic acid) having been previously infused into the reference sprayer at 300 nL/min for this. The default parameters were used for peak picking and alignment. The peptide identification was performed against Uniprot human FASTA sequences (release 2018_04). A chaperone protein ClpB (ClpB) protein sequence (CLPB_ECOLI (P63285)) was inserted for label‐free quantification. “Fixed modification” at cysteine (carbamidomethyl) and “variable” at methionine (oxidation) were used. One missed cleavage for trypsin was allowed. The automatic settings for the fragment and peptide error tolerances were used, while the false discovery rate (FDR) was set to less than 2%. The default parameters for ion matching were used, which are as follows: one or more ion fragments per peptide, three or more fragments per protein, and one or more peptides per protein.

The proteins were grouped according to the parsimony principle, although it is known that due to over‐stringency, Progenesis QI for proteomics does not follow a strict parsimonious approach.^26^ Therefore, if two proteins are found that share common peptides, the protein with fewer peptides will be subsumed into the protein with more peptides. If the coverages of two or more proteins are equal, all the relevant proteins will be listed under the lead protein that has the highest coverage/score. In this study, quantification was performed using the data of the lead identity peptide. For further details, see Nonlinear Dynamics’ website (www.nonlinear.com).

### 2.5 Further analysis

The differences between the groups were analyzed using the ANOVA test and p‐values were corrected using the Bonferroni correction. Bonferroni‐corrected ANOVA‐passing p‐values of less than 0.01 were considered significant to ensure stringent analysis. Data were normalized by Pareto scaling, and hierarchical clustering and principal component analysis were performed using Metaboanalyst, version 4.0.^27^, ^28^ The feature “autoscaling” was further used during hierarchical clustering to generate heatmaps. Pathway analysis was performed with Ingenuity Pathway Analysis (IPA, build version 486068M, content version 46 901 286, QIAGEN Bioinformatics).^29^ All proteins that passed the cutoff of an ANOVA *P*‐value of less than .05 were used for pathway analysis. Pathway analysis was performed separately for all ANOVA‐passing proteins for all samples, stage II samples, and stage III samples between two tumor locations at a time (right colon/left colon, right colon/rectum, and left colon/rectum), as IPA cannot perform comparisons between three groups simultaneously. The mass spectrometry proteomics data have been deposited to the ProteomeXchange Consortium via the PRIDE^30^, ^31^ partner repository with the dataset identifier PXD013150 and 10.6019/PXD013150.

## 3 RESULTS

### 3.1 Protein identification and analysis

In this study, we analyzed plasma samples from 83 CRC patients that were divided into groups based on primary tumor location (right colon, left colon, rectum). Detailed patient characteristics are given in Table S1. The samples were also divided into groups based on location and tumor stage (II or III). Twenty‐seven patients had tumors in the right colon, 26 in the left colon, and 30 in the rectum. We quantified 224 proteins that contained two or more unique peptides and these 224 proteins were used for further analysis. These proteins with relevant data are given in Table S2.

### 3.2 All samples

When all plasma samples regardless of tumor stage were compared according to tumor location, 125 proteins passed the cut‐off of a Bonferroni‐corrected ANOVA *P*‐value of less than .01. The top 20 proteins according to fold change are given in Table 1, and all 125 proteins are given in Table S3A. The largest differences were seen between plasma protein expression in samples from patients with cancer in the right colon and rectum. Levels of keratins, type I cytoskeletal 16 (*KRT16*), 9 (*KRT9*), and 10 (*KRT10*), as well as complement factor H‐related protein 4 (*CFHR4*) and 1 (*CFHR1*), had higher levels in plasma samples from patients with cancer in the right colon (with fold changes between 6.2‐13.0). The plasma levels of long‐chain‐fatty‐acid‐‐CoA ligase 5 (*ACSL5*, fold change of 6.3), an enzyme, were also higher in plasma samples from patients with cancer in the right colon.

**TABLE 1:** Top 20 plasma proteins with a Bonferroni‐corrected *P*‐value of less than .01 when all samples were compared

Accession	Peptide count	Unique peptides	Confidence score	ANOVA (*P*)	Bonferroni‐corrected *P*‐values	Max fold change	Power	Highest mean condition	Lowest mean condition	AUC (right colon/left colon)	AUC (right colon/rectum)	AUC (left colon/rectum)	Protein name	Gene name
Q92496	6	2	35.6	<.0001	<.0001	13.0	1.00	Right colon	Rectum	0.67	0.97	0.94	Complement factor H‐related protein 4	CFHR4
P08779;P02533	3	2	15.5	1.33E‐15	2.98E‐13	11.4	1.00	Right colon	Rectum	0.72	0.98	0.95	Keratin_ type I cytoskeletal 16	KRT16
P35527	6	5	28.7	<.0001	<.0001	10.1	1.00	Right colon	Rectum	0.73	1.00	0.98	Keratin_ type I cytoskeletal 9	KRT9
P13645;Q7Z3Y7;Q7Z3Y8;Q7Z3Y9;Q7Z3Z0	6	5	36.0	<.0001	<.0001	7.8	1.00	Right colon	Rectum	0.61	0.99	0.98	Keratin_ type I cytoskeletal 10	KRT10
Q9ULC5	5	3	23.0	<.0001	<.0001	6.3	1.00	Right colon	Rectum	0.85	1.00	0.98	Long‐chain‐fatty‐acid‐‐CoA ligase 5	ACSL5
Q03591	15	2	131.6	3.69E‐12	8.27E‐10	6.2	1.00	Right colon	Rectum	0.62	0.94	0.90	Complement factor H‐related protein 1	CFHR1
P04264	7	2	39.0	2.98E‐09	6.68E‐07	5.3	1.00	Left colon	Rectum	0.54	0.90	0.88	Keratin_ type II cytoskeletal 1	KRT1
Q9UIF8	4	2	19.5	<0.0001	<0.0001	4.6	1.00	Right colon	Rectum	0.68	0.95	0.91	Bromodomain adjacent to zinc finger domain protein 2B	BAZ2B
Q3V6T2	15	6	70.1	<0.0001	<0.0001	4.3	1.00	Right colon	Rectum	0.64	1.00	0.99	Girdin	CCDC88A
A6NMZ7;P51570;Q13158;Q6NT55	5	2	24.3	<.0001	<.0001	4.3	1.00	Right colon	Rectum	0.80	1.00	0.97	Collagen alpha‐6(VI) chain	COL6A6
H3BMM5	3	2	13.7	<.0001	<.0001	4.2	1.00	Right colon	Rectum	0.60	0.98	0.97	Uncharacterized protein	
P00746	3	2	12.0	<.0001	<.0001	3.8	1.00	Right colon	Rectum	0.61	0.97	0.97	Complement factor D	CFD
Q86UX7	7	2	37.3	8.29E‐11	1.86E‐08	3.8	1.00	Right colon	Rectum	0.71	0.94	0.86	Fermitin family homolog 3	FERMT3
Q12805	9	8	51.7	<.0001	<.0001	3.4	1.00	Right colon	Rectum	0.79	1.00	0.96	EGF‐containing fibulin‐like extracellular matrix protein 1	EFEMP1
O43866	7	5	38.1	<.0001	<.0001	3.4	1.00	Right colon	Rectum	0.63	0.99	0.95	CD5 antigen‐like	CD5L
B1AJZ9	10	3	49.0	3.89E‐15	8.70E‐13	3.4	1.00	Right colon	Rectum	0.71	0.96	0.92	Forkhead‐associated domain‐containing protein 1	FHAD1
O75038	5	3	20.9	1.11E‐16	2.49E‐14	3.3	1.00	Right colon	Rectum	0.72	0.97	0.90	1‐phosphatidylinositol 4_5‐bisphosphate phosphodiesterase eta‐2	PLCH2
Q9P2E3	9	2	55.8	4.80E‐09	1.07E‐06	3.1	1.00	Right colon	Rectum	0.50	0.85	0.90	NFX1‐type zinc finger‐containing protein 1	ZNFX1
Q6UB98	6	2	36.6	<.0001	<.0001	3.0	1.00	Right colon	Rectum	0.72	0.98	0.95	Ankyrin repeat domain‐containing protein 12	ANKRD12
Q9Y4C1	4	3	21.0	<.0001	<.0001	2.9	1.00	Right colon	Rectum	0.78	0.99	0.96	Lysine‐specific demethylase 3A	KDM3A

Note

The list with all significantly different proteins can be found in Table S3A.

The area under the curve (AUC) values was also calculated for the 125 proteins passing the cutoff of a Bonferroni‐corrected ANOVA *P*‐value of less than .01 when all samples were compared. This was done by comparing the proteins between two tumor locations at a time (right colon/left colon, right colon/rectum, and left colon/rectum). The results are given in Table S3A and further strengthen our findings that plasma protein expression is significantly different between patients with cancer in the colon and rectum, with multiple proteins reaching AUC values of > 0.9, increasing their confidence. The fold changes for all proteins between two tumor locations only (right colon/left colon, right colon/rectum, and left colon/rectum) are given for reference in Table S4A.

Pareto scaling was performed and principal component analysis (PCA) biplots and hierarchical clustering heatmaps were generated. The PCA when all proteins were considered is given in Figure S1. The heatmap when only proteins that passed the cutoff of a Bonferroni‐corrected ANOVA *P*‐value of less than .01 were considered is given in Figure S2. Figure S2 shows that samples from patients with rectal cancer cluster together, although a few of these samples clustered together with samples from patients with cancer in the left colon.

### 3.3 Stage II samples

The samples from patients with stage II CRC only were also analyzed according to tumor location, and 46 proteins passed the cut‐off of a Bonferroni‐corrected ANOVA *P*‐value of less than .01 (Table S3B). The top 20 proteins according to fold change are shown in Table 2. Again, the greatest differences were seen between samples from patients with stage II cancer in the right colon or rectum. The protein with the largest fold change (10.7) was KRT9, with higher levels in samples from patients with cancer in the left colon compared to the rectum. CFHR4 (fold change of 8.4) had higher levels in samples from patients with cancer in the right colon compared to the rectum. The fold changes for all proteins compared between two tumor locations only are given in Table S4B.

**TABLE 2:** Top 20 plasma proteins with a Bonferroni‐corrected *P*‐value of less than .01 when only stage II samples were compared

Accession	Peptide count	Unique peptides	Confidence score	ANOVA (*P*)	Bonferroni‐corrected *P*‐values	Max fold change	Power	Highest mean condition	Lowest mean condition	Protein name	Gene name
P35527	6	5	28.7	7.95E‐08	1.78E‐05	10.7	1.00	Left colon stage II	Rectum stage II	Keratin_ type I cytoskeletal 9	KRT9
Q92496	6	2	35.6	7.17E‐06	1.61E‐03	8.4	1.00	Right colon stage II	Rectum stage II	Complement factor H‐related protein 4	CFHR4
P08779;P02533	3	2	15.5	9.54E‐06	2.14E‐03	6.1	1.00	Right colon stage II	Rectum stage II	Keratin_ type I cytoskeletal 16	KRT16
P13645;Q7Z3Y7;Q7Z3Y8;Q7Z3Y9;Q7Z3Z0	6	5	36.0	8.28E‐09	1.86E‐06	5.6	1.00	Left colon stage II	Rectum stage II	Keratin_ type I cytoskeletal 10	KRT10
Q3V6T2	15	6	70.1	2.01E‐10	4.50E‐08	4.6	1.00	Right colon stage II	Rectum stage II	Girdin	CCDC88A
Q9ULC5	5	3	23.0	9.65E‐14	2.16E‐11	4.6	1.00	Right colon stage II	Rectum stage II	Long‐chain‐fatty‐acid‐‐CoA ligase 5	ACSL5
P00746	3	2	12.0	3.12E‐09	6.98E‐07	4.3	1.00	Right colon stage II	Rectum stage II	Complement factor D	CFD
O43866	7	5	38.1	1.97E‐10	4.42E‐08	4.0	1.00	Right colon stage II	Rectum stage II	CD5 antigen‐like	CD5L
A6NMZ7;P51570;Q13158;Q6NT55	5	2	24.3	1.94E‐11	4.33E‐09	4.0	1.00	Right colon stage II	Rectum stage II	Collagen alpha‐6(VI) chain	COL6A6
Q9UIF8	4	2	19.5	2.24E‐06	5.01E‐04	3.8	1.00	Right colon stage II	Rectum stage II	Bromodomain adjacent to zinc finger domain protein 2B	BAZ2B
Q92777	5	3	27.1	2.41E‐05	5.40E‐03	3.5	1.00	Right colon stage II	Rectum stage II	Synapsin‐2	SYN2
H3BMM5	3	2	13.7	5.10E‐08	1.14E‐05	3.5	1.00	Right colon stage II	Rectum stage II	Uncharacterized protein	
Q96PZ0	3	2	21.0	2.54E‐05	5.70E‐03	3.2	1.00	Right colon stage II	Rectum stage II	Pseudouridylate synthase 7 homolog	PUS7
Q12805	9	8	51.7	1.81E‐11	4.04E‐09	3.2	1.00	Right colon stage II	Rectum stage II	EGF‐containing fibulin‐like extracellular matrix protein 1	EFEMP1
B1AJZ9	10	3	49.0	1.06E‐06	2.38E‐04	3.1	1.00	Right colon stage II	Rectum stage II	Forkhead‐associated domain‐containing protein 1	FHAD1
Q9Y4C1	4	3	21.0	6.88E‐09	1.54E‐06	3.0	1.00	Right colon stage II	Rectum stage II	Lysine‐specific demethylase 3A	KDM3A
O75038	5	3	20.9	1.99E‐07	4.46E‐05	2.9	1.00	Right colon stage II	Rectum stage II	1‐phosphatidylinositol 4_5‐bisphosphate phosphodiesterase eta‐2	PLCH2
Q9UK55	22	13	125.8	3.53E‐05	7.92E‐03	2.9	1.00	Right colon stage II	Rectum stage II	Protein Z‐dependent protease inhibitor	SERPINA10
Q8IV77	3	2	14.4	4.37E‐07	9.78E‐05	2.9	1.00	Right colon stage II	Rectum stage II	Cyclic nucleotide‐gated cation channel alpha‐4	CNGA4
P04070	6	4	29.2	4.30E‐07	9.63E‐05	2.7	1.00	Right colon stage II	Rectum stage II	Vitamin K‐dependent protein C	PROC

Note

The list with all significantly different proteins can be found in Table S3B.

**FIGURE 1:**
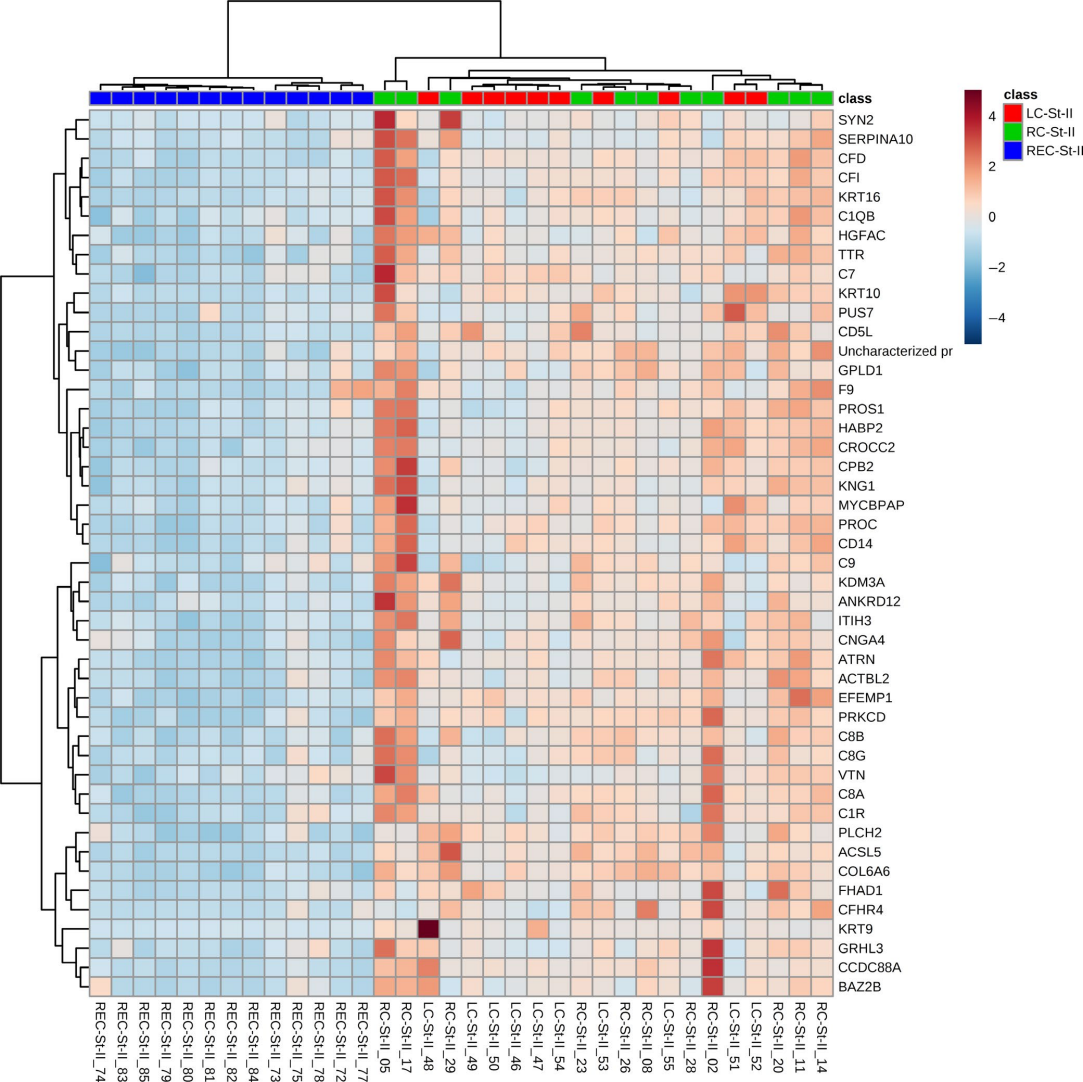
Hierarchical clustering heatmap of Pareto‐scaled proteins using only those proteins that passed the cutoff of Bonferroni‐corrected ANOVA *P*‐value of less than .01 when only stage II samples were compared. The heatmap shows that plasma samples from patients with colon and rectal cancer form distinct clusters. Additionally, samples from patients with tumors in the right or left colon also showed a tendency to cluster together, although with some overlap between these colon tumors

PCA biplots and hierarchical clustering heatmaps were generated using stage II samples only with Pareto‐scaled data. The PCA when all proteins were considered is given in Figure S3. The heatmap when only the proteins that passed the cut‐off of a Bonferroni‐corrected ANOVA *P*‐value of less than .01 were considered is given in Figure 1. As seen in Figure 1, the plasma samples from patients with rectal cancer cluster together, while samples from patients with colon cancer form a separate cluster. While there is a tendency of samples from patients with tumors in the right or left colon to cluster together depending on tumor location, some overlap can be seen between the colon tumors.

### 3.4 Stage III samples

When plasma samples from patients with stage III CRC were compared according to tumor location, 92 proteins passed the cut‐off of a Bonferroni‐corrected ANOVA *P*‐value of less than .01 (Table S3C). The top 20 proteins according to fold change are shown in Table 3. The top three proteins, KRT16 (with a fold change of 45.6), CFHR4 (with a fold change of 41.2), and CFHR1 (with a fold change of 27.9), all displayed higher plasma levels in samples from patients with stage III cancer in the right colon than the rectum. The fold changes for all proteins between two tumor locations only are given in Table S4C.

**TABLE 3:** Top 20 plasma proteins with a Bonferroni‐corrected *P*‐value of less than .01 when only stage III samples were compared

Accession	Peptide count	Unique peptides	Confidence score	ANOVA (*P*)	Bonferroni‐corrected *P*‐values	Max fold change	Power	Highest mean condition	Lowest mean condition	Protein name	Gene name
P08779;P02533	3	2	15.5	1.83E‐14	4.10E‐12	45.6	1.00	Right colon stage III	Rectum stage III	Keratin_ type I cytoskeletal 16	KRT16
Q92496	6	2	35.6	1.52E‐14	3.41E‐12	41.2	1.00	Right colon stage III	Rectum stage III	Complement factor H‐related protein 4	CFHR4
Q03591	15	2	131.6	1.67E‐12	3.73E‐10	27.9	1.00	Right colon stage III	Rectum stage III	Complement factor H‐related protein 1	CFHR1
P35527	6	5	28.7	5.55E‐14	1.24E‐11	18.2	1.00	Right colon stage III	Rectum stage III	Keratin_ type I cytoskeletal 9	KRT9
P13645;Q7Z3Y7;Q7Z3Y8;Q7Z3Y9;Q7Z3Z0	6	5	36.0	<.0001	<.0001	15.2	1.00	Right colon stage III	Rectum stage III	Keratin_ type I cytoskeletal 10	KRT10
Q9ULC5	5	3	23.0	<.0001	<.0001	9.5	1.00	Right colon stage III	Rectum stage III	Long‐chain‐fatty‐acid‐‐CoA ligase 5	ACSL5
Q9UIF8	4	2	19.5	1.18E‐12	2.64E‐10	5.9	1.00	Right colon stage III	Rectum stage III	Bromodomain adjacent to zinc finger domain protein 2B	BAZ2B
H3BMM5	3	2	13.7	<.0001	<.0001	5.8	1.00	Left colon stage III	Rectum stage III	Uncharacterized protein	
P04264	7	2	39.0	1.04E‐07	2.33E‐05	5.6	1.00	Right colon stage III	Rectum stage III	Keratin_ type II cytoskeletal 1	KRT1
Q86UX7	7	2	37.3	1.05E‐08	2.36E‐06	5.4	1.00	Right colon stage III	Rectum stage III	Fermitin family homolog 3	FERMT3
A6NMZ7;P51570;Q13158;Q6NT55	5	2	24.3	1.11E‐16	2.49E‐14	4.6	1.00	Right colon stage III	Rectum stage III	Collagen alpha‐6(VI) chain	COL6A6
Q6UB98	6	2	36.6	<.0001	<.0001	4.3	1.00	Right colon stage III	Rectum stage III	Ankyrin repeat domain‐containing protein 12	ANKRD12
Q3V6T2	15	6	70.1	1.01E‐13	2.25E‐11	4.1	1.00	Right colon stage III	Rectum stage III	Girdin	CCDC88A
B1AJZ9	10	3	49.0	3.69E‐11	8.27E‐09	3.8	1.00	Right colon stage III	Rectum stage III	Forkhead‐associated domain‐containing protein 1	FHAD1
Q12805	9	8	51.7	4.45E‐14	9.97E‐12	3.8	1.00	Right colon stage III	Rectum stage III	EGF‐containing fibulin‐like extracellular matrix protein 1	EFEMP1
O75038	5	3	20.9	2.17E‐10	4.86E‐08	3.7	1.00	Right colon stage III	Rectum stage III	1‐phosphatidylinositol 4_5‐bisphosphate phosphodiesterase eta‐2	PLCH2
P00746	3	2	12.0	2.38E‐09	5.32E‐07	3.4	1.00	Right colon stage III	Rectum stage III	Complement factor D	CFD
Q8N841	6	2	26.9	3.14E‐06	7.02E‐04	3.4	1.00	Right colon stage III	Rectum stage III	Tubulin polyglutamylase TTLL6	TTLL6
Q92698	3	2	16.5	3.93E‐09	8.81E‐07	3.3	1.00	Left colon stage III	Rectum stage III	DNA repair and recombination protein RAD54‐like	RAD54L
P59047	4	3	21.8	5.08E‐06	1.14E‐03	3.3	1.00	Right colon stage III	Rectum stage III	NACHT_ LRR and PYD domains‐containing protein 5	NLRP5

Note

The list with all significantly different proteins can be found in Table S3C.

**FIGURE 2:**
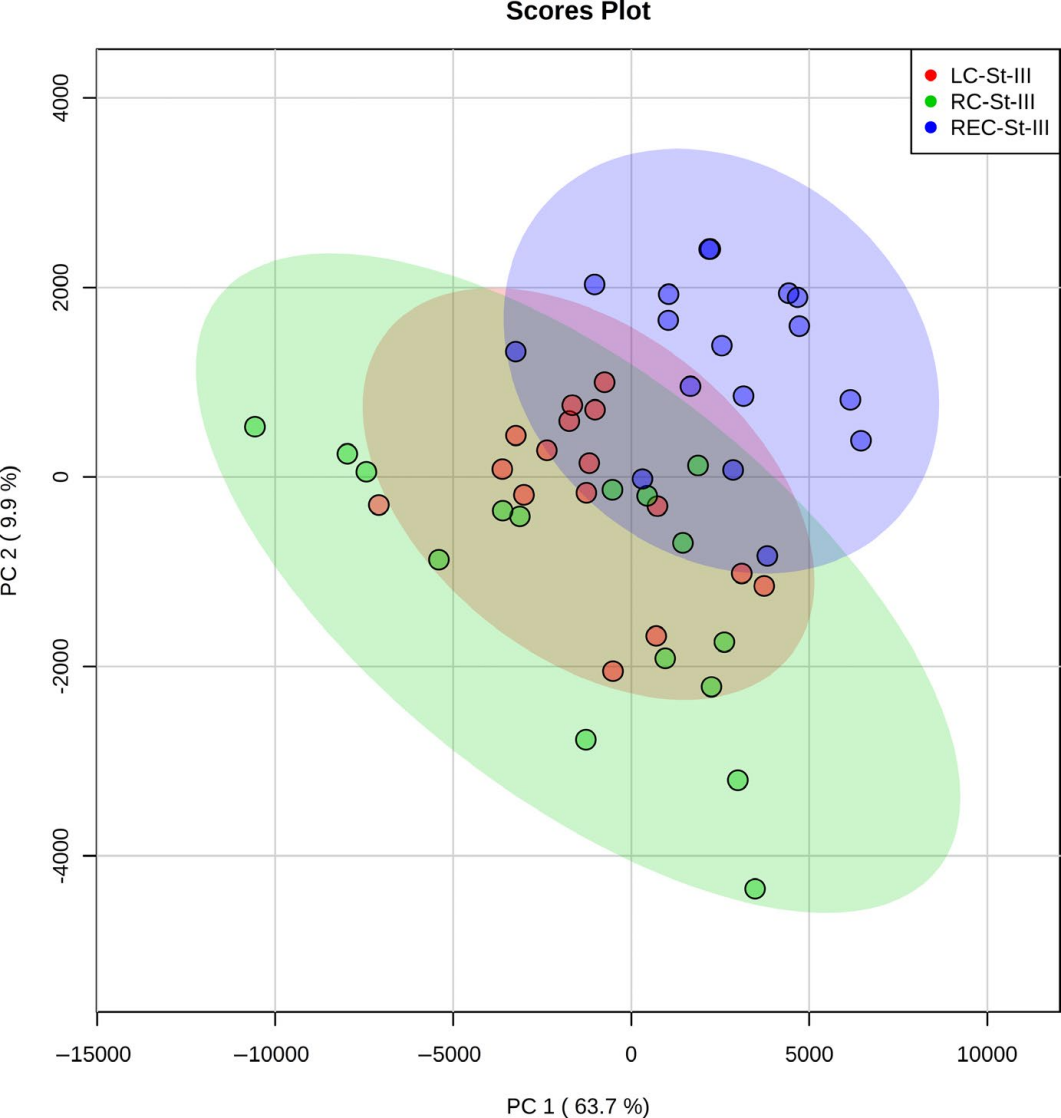
PCA biplot showing stage III samples only when all Pareto‐scaled proteins were considered

**FIGURE 3:**
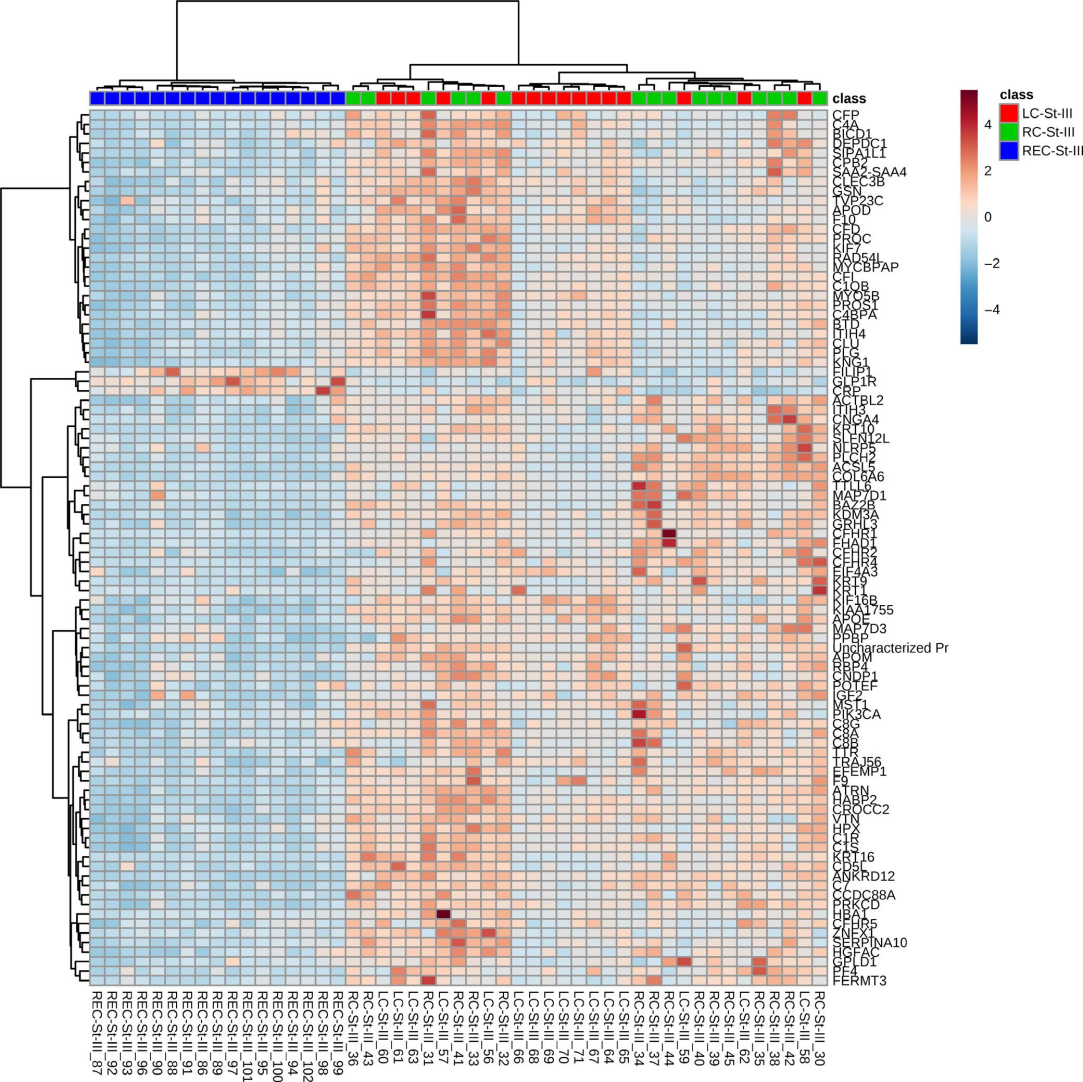
Hierarchical clustering heatmap of Pareto‐scaled proteins using only those proteins that passed the cutoff of Bonferroni‐corrected ANOVA *P*‐value of less than .01 when only stage III samples were compared. This heatmap also shows that plasma samples from patients with colon and rectal cancer form distinct clusters, and that samples from patients with right‐ or left‐sided colon tumors also form separate clusters, to some extent

PCA biplots and hierarchical clustering heatmaps were generated using Pareto‐scaled data. The PCA when all proteins were considered is given in Figure 2 and shows that plasma samples from patients with rectal cancer mostly separate from plasma samples from patients with right‐ or left‐sided colon cancer, which overlap more. The heatmap when only the proteins that passed the cut‐off of a Bonferroni‐corrected ANOVA p‐value of less than 0.01 were considered were considered is given in Figure 3, and similar to when only stage II samples were mapped, shows that samples from patients with rectal cancer form a distinct group. The plasma samples from patients with stage III tumors in the right or left colon also had a tendency to separate, although some overlap was seen (Figure 3).

### 3.5 Pathway analysis

When the ANOVA‐passing proteins for all samples were analyzed by IPA between two tumor locations (right colon/left colon, right colon/rectum, and left colon/rectum) at a time, the top five canonical pathways enriched in all groups were LXR/RXR activation, acute phase response signaling, the complement system, FXR/RXR activation, and the coagulation system (Figure S4). The same analysis was carried out for stage II samples, and the top five canonical pathways in all comparisons were the complement system, acute phase response signaling, LXR/RXR activation, FXR/RXR activation, and the coagulation system (Figure S5). For stage III samples, the top five canonical pathways enriched in all comparisons were LXR/RXR activation, the complement system, acute phase response signaling, FXR/RXR activation, and the coagulation system (Figure S6).

## 4 DISCUSSION

In this study, we observed widespread differences in plasma protein expression depending on primary tumor location, both when samples were analyzed regardless of and according to tumor stage (II or III). In all three groups (all samples, stage II only, and stage III only), the plasma levels of CFHR4 (AUC of 0.97 when samples from patients with cancer in the right colon and rectum were compared) and ACSL5 (AUC of 1 between these samples) were found to be much higher in samples from patients with cancer in the right colon compared to the rectum (Table S3). In samples from patients with stage III cancer, levels of CFHR4 were over 40 times higher in samples from patients with cancer in the right colon (Table S3C). Levels of CFHR1 were also significantly higher in the same samples when all and only stage III samples were compared (Table S3A,C). The expression of complement system components is increased in cancer, and activation of the complement system has been shown to promote tumor growth in the context of inflammation.^32^, ^33^ Our findings therefore indicate that inflammation may be more important in driving carcinogenesis in the right colon than rectum.

The biggest differences in plasma protein expression were seen between samples from patients with cancer in the right colon compared to the rectum (Table S3), an understandable finding, as these locations are anatomically the furthest from each other. However, there were also significant differences in plasma protein expression between samples from patients with cancer in the right and left colon (Table S4). Tumors in the right and left colon follow separate pathways of carcinogenesis as they display different molecular features. Right‐sided tumors more frequently display MSI and CIMP, while left‐sided tumors are more often characterized by chromosomal instability and mutations in genes such as *TP53*.^4^, ^34^ Additionally, tumors in the right colon also tend to display an increased infiltration of immune cells compared to tumors in the left colon, something which may have contributed to the differences in plasma protein expression, such as between complement components, observed in this study (Table S3).^35^

Pathway analysis by IPA found multiple canonical pathways to be enriched in this dataset, with the top five pathways enriched being the same regardless of tumor location or stage (Figures S4‐S6). The enrichment of pathways such as LXR/RXR and FXR/RXR activation point to altered lipid metabolism, as LXR/RXR and FXR/RXR heterodimers have important roles in lipid and bile acid metabolism.^36^, ^37^, ^38^ The observed enrichment of pathways involved in lipid metabolism may be affected by factors such as bile acid concentration, which differs between the right and left colon.^39^ Higher levels of ACSL5 were seen in samples from patients with cancer in the right colon compared to the rectum when all samples were analyzed (AUC of 1; Table 1). ACSL5 is an enzyme involved in lipid metabolism,^40^ and the differences in ACSL5 levels may have contributed to the enrichment of pathways such as LXR/RXR and FXR/RXR activation seen (Fig. S4B). The enrichment of pathways such as acute phase response signaling and the complement system indicate inflammation as having different roles in CRC depending on tumor location, something that is further supported by our findings that plasma levels of proteins such as complement differ depending on tumor location (Table S3).

In this study, the plasma proteomic profiles of patients with rectal cancer were found to be significantly different from those of patients with colon cancer. It has been suggested that colon and rectal tumors follow separate pathways of carcinogenesis due to the different mutations commonly seen in colon and rectal cancer, indicating that they select for mutations in distinct signaling pathways.^14^ The results of a study by Kapiteijn et al^16^ indicated that the p53 pathway is more important in rectal than colon cancer. Their study also found that rectal tumors were more often positive for nuclear ß‐catenin than colon tumors, although this finding was not associated with the presence of a mutation in the adenomatous polyposis coli (*APC*) gene. Another study found that mutations in the KRAS proto‐oncogene (*KRAS*) were more commonly detected in colon tumors than rectal tumors, and that the number of mutations detected was higher in colon tumors when compared to rectal tumors, further strengthening the theory that the pathways to carcinogenesis differ for colon and rectal tumors.^41^ Different pathways to carcinogenesis and the presence of different mutations in colon and rectal tumors likely affects plasma protein expression. This may help explain the differences in plasma protein profiles between samples from patients with cancer in the colon and rectum seen in the current study.

Previous mass spectrometric studies have mainly focused on identifying new proteins of use for the diagnosis and early detection of CRC and have focused on comparing samples from CRC patients and healthy controls.^42^, ^43^, ^44^ Several studies have investigated differences in protein expression between CRC in different locations and studied the expression of specific proteins in tissue samples using immunohistochemistry.^45^, ^46^, ^47^, ^48^, ^49^ In this study, we chose to analyze plasma samples from CRC patients only, without the inclusion of healthy controls, due to the paucity of such studies. A couple of recent studies have further investigated the differences between right‐ and left‐sided colon cancer. One study using plasma metabolomic profiling found significant differences between right‐ and left‐sided colon cancer, with six metabolites identified as potential biomarkers for tumor location.^50^ A comparative proteogenomic study found distinct mutations and proteins between right‐sided colon cancer, left‐sided colon cancer, and rectal cancer.^51^ Another study investigated plasma protein expression during CRC progression from stage II to III and showed that there are both differences and overlap in plasma protein expression during cancer progression.^21^ However, this study did not compare plasma protein profiles depending solely on primary tumor location, which is the focus of the current study.

The aim of this study was to establish if plasma protein expression differed in a tumor location‐specific manner. This pilot study did not aim to identify candidates for new biomarkers for the diagnosis of CRC, as the current methods of diagnosis, involving colonoscopy and biopsy, are more specific than measuring the levels of plasma proteins. The plasma proteins identified in this study were also not tumor‐derived and are therefore non‐specific for CRC, as their concentrations can be elevated due to other factors and conditions than CRC. This decreases their value as diagnostic markers, and their diagnostic value has subsequently not been validated.

The results of this study show that plasma protein expression is distinct depending on primary tumor location and can clearly classify colon and rectal tumors, and, to a lesser extent, right‐ and left‐sided colon tumors. This study was strengthened by the relatively large number of samples analyzed, although it was limited by the lack of CRC tumor tissues studied simultaneously, which would have enabled the comparison of plasma protein expression with tissue protein expression. In future studies, we aim to analyze and compare protein expression in tumor tissue samples based on location, as well as further investigate the proteins identified here. The findings presented in this study will help to further define colon and rectal tumors (and to a lesser extent, right‐ and left‐sided colon tumors) as separate entities as shown by the widespread differences in plasma protein profile and dysregulated pathways. Additionally, they also provide a basis for future studies aiming to continue the investigation of the distinct disease entities that constitute CRC.

## CONFLICT OF INTEREST

The authors declare no conflict of interest.

## AUTHORS’ CONTRIBUTIONS

MH, SJ, MS, AR, RR, and CH conceived and designed the study. MH and CH collected the plasma samples as well as the patients’ clinical data. MH, SJ, and TT acquired the mass spectrometric data. MH, SJ, and MS analyzed and interpreted the data. MH wrote the manuscript. All authors revised the manuscript. AR, RR, and CH provided resources.

## Ethics approval and consent to participate

Written informed consent was obtained from all patients prior to sample collection. This study was approved by the Surgical Ethics Committee of Helsinki University Hospital (Dnro HUS 226/E6/06, extension TMK02 §66 17.4.2013). All research was performed in accordance with the Declaration of Helsinki.

## Supporting information

Fig S1‐S6

Table S1

Table S2

Table S3

Table S4

## Data Availability

The mass spectrometry proteomics data generated during the current study are available at the ProteomeXchange Consortium via the PRIDE partner repository with the dataset identifier PXD013150 and 10.6019/PXD013150.
