# Supplementary material for: Plasma protein expression differs between colorectal cancer patients depending on primary tumor location
Source: Cancer Med. 2020 May 26;9(14):5221–34. doi: 10.1002/cam4.3178 (PMC7367633; doi:10.1002/cam4.3178)
Supplement: Supplementary file 1 — Fig S1‐S6 [file CAM4-9-5221-s001.pdf]

Supplementary Figures

Plasma protein expression differs between colorectal cancer patients depending on primary tumor location

Matilda Holm[1,2,3,4,*], Sakari Joenväärä[5,6], Mayank Saraswat[5,6,7], Tiialotta Tohmola[5,6,8], Ari Ristimäki[2,4,6], Risto Renkonen[5,6,¶], Caj Haglund[1,3,6,¶]

[1]Department of Surgery, Faculty of Medicine, University of Helsinki and Helsinki University Hospital, Helsinki, Finland
[2]Department of Pathology, Faculty of Medicine, University of Helsinki and Helsinki University Hospital, Helsinki, Finland
[3]Translational Cancer Medicine Research Program, Faculty of Medicine, University of Helsinki, Helsinki, Finland
[4]Applied Tumor Genomics Research Program, Faculty of Medicine, University of Helsinki, Helsinki, Finland
[5]Transplantation Laboratory, Haartman Institute, University of Helsinki, Helsinki, Finland
[6]HUSLAB, Helsinki University Hospital, Helsinki, Finland
[7]Department of Laboratory Medicine and Pathology, Mayo Clinic, Rochester, Minnesota, USA
[8]Department of Biosciences, Faculty of Biological and Environmental Sciences, University of Helsinki, Helsinki, Finland

¶equal contribution

*Corresponding author
Mail to: matilda.holm@helsinki.fi (MH)

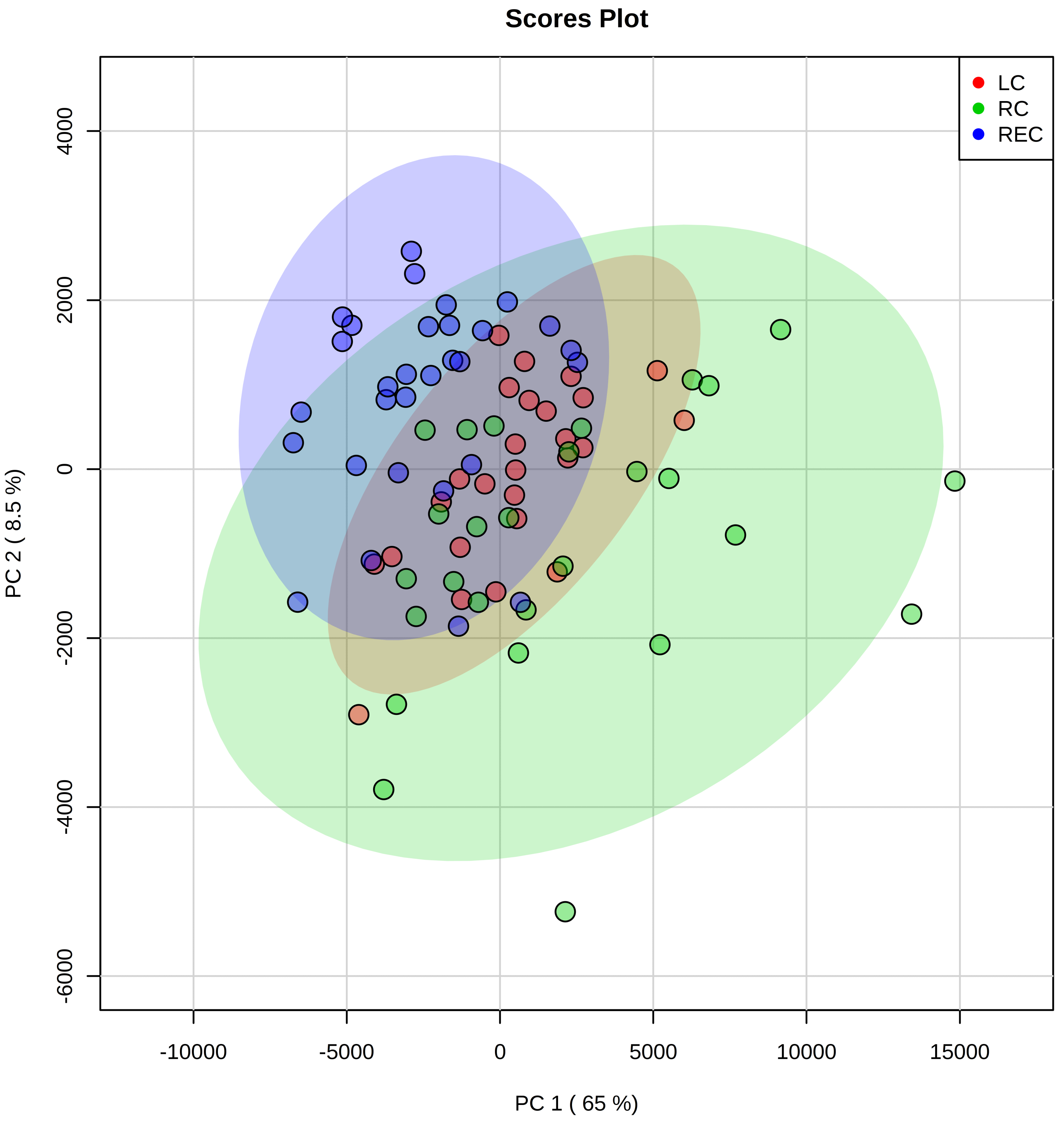

Supplementary Figure S1 – The PCA biplot showing all samples when all Pareto-scaled proteins were considered.

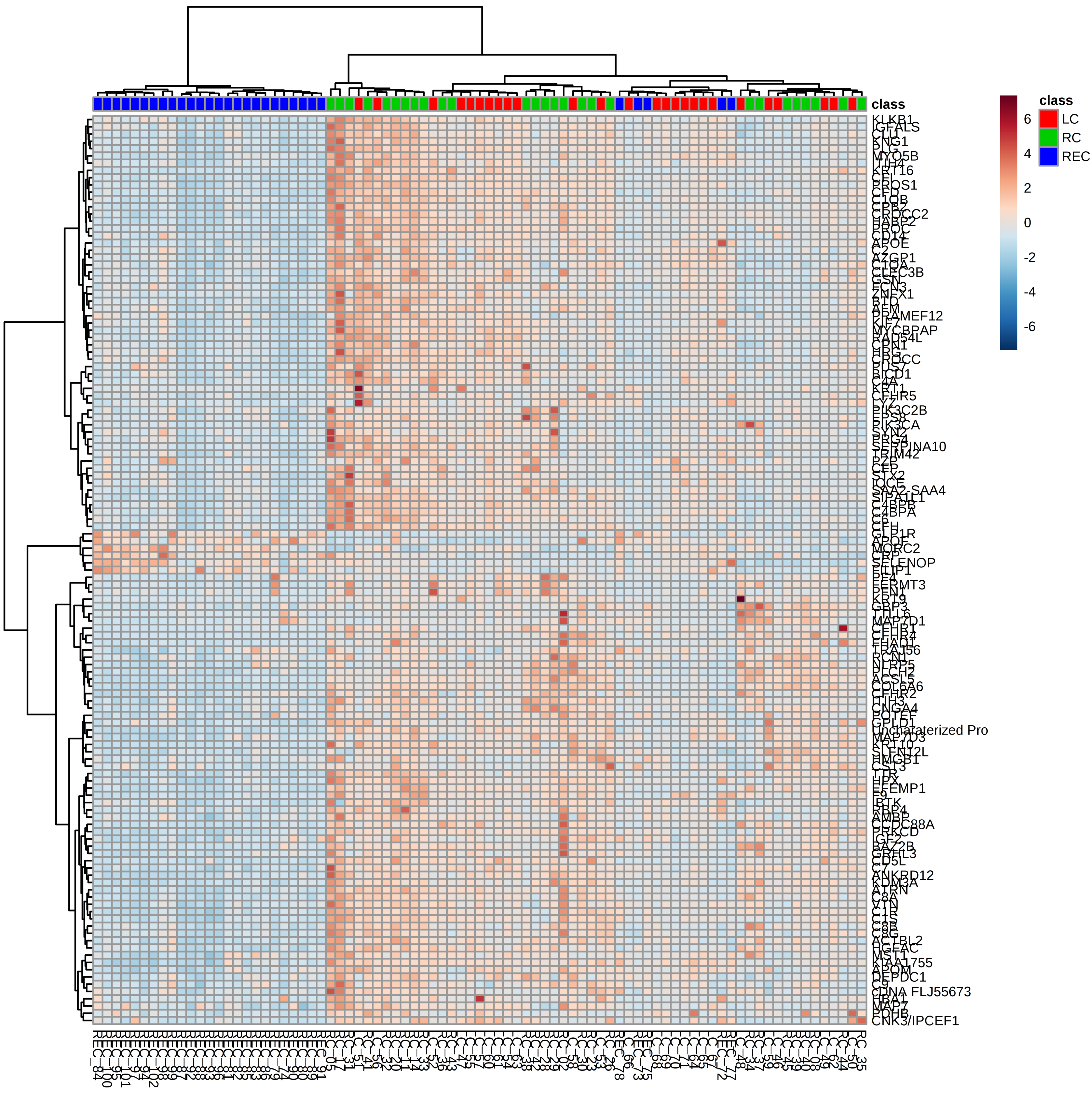

Supplementary Figure S2 – The hierarchical clustering heatmap of Pareto-scaled proteins using only those proteins that passed the cutoff of Bonferroni-corrected ANOVA p-value of less than 0.01. Plasma samples from patients with rectal cancer form a distinct cluster, with some samples clustering together with samples from patients with cancer in the left colon.

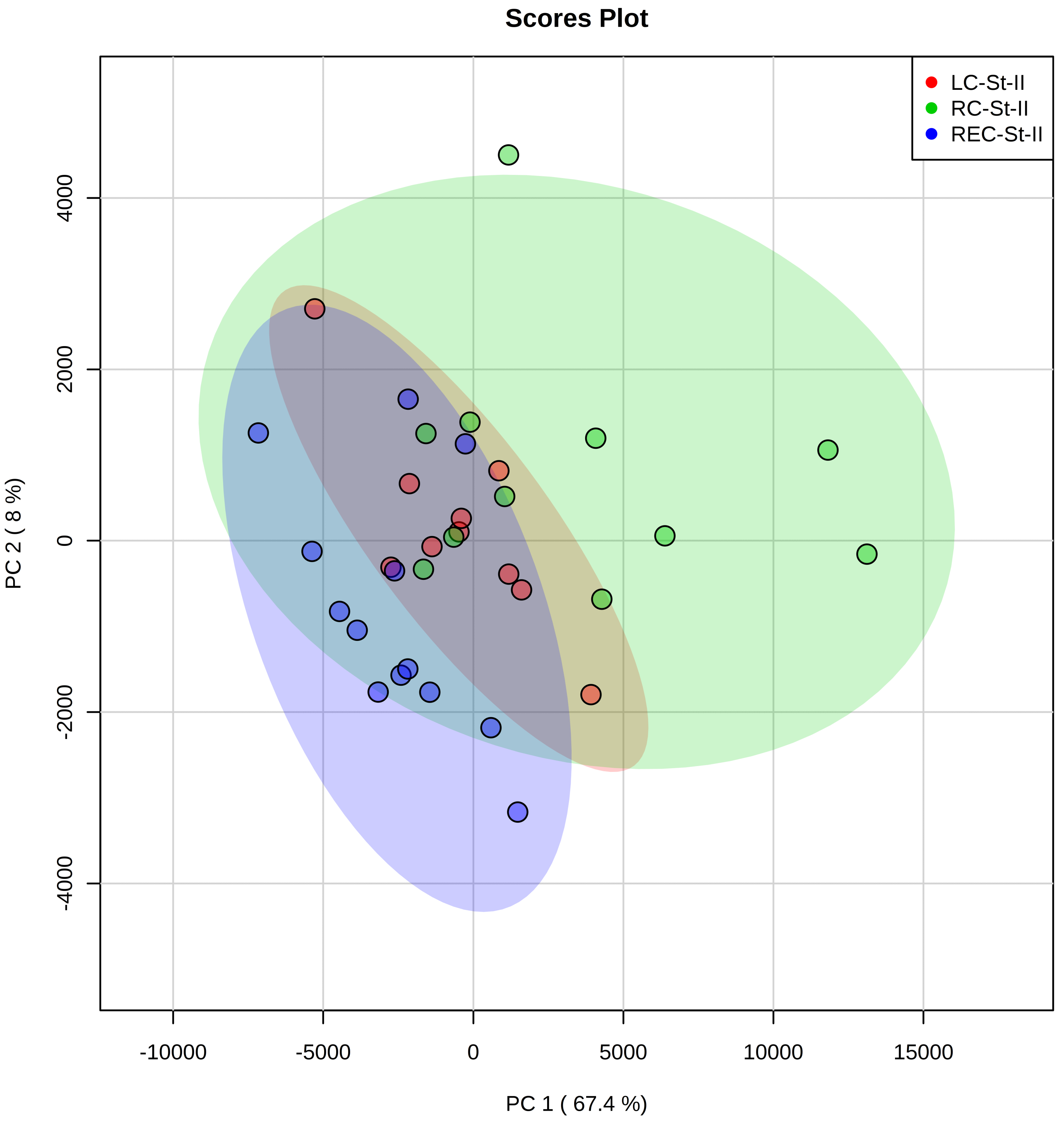

Supplementary Figure S3 – The PCA biplot showing only stage II samples when all Pareto-scaled proteins were considered.

Supplementary Figure S4 – The top 10 canonical pathways found by IPA when all samples were analyzed. Supplementary Figure S4A shows thecanonical pathways enriched when samples from patients with cancer in the right or left colon were analyzed. Supplementary Figure S4B shows the pathways enriched when samples from patients with cancer in the right colon or rectum were analyzed. Supplementary FigureS4C shows the pathways enriched when samples from patients with cancer in the left colon or rectum were analyzed.

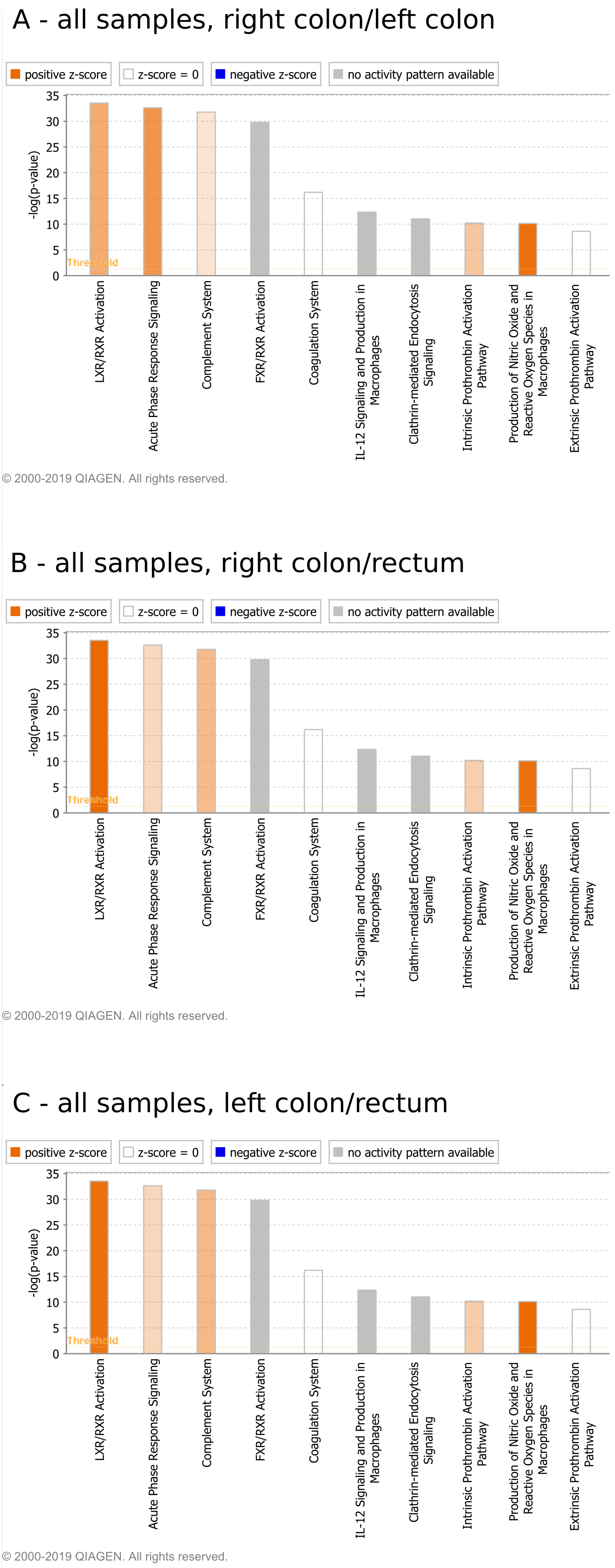

Supplementary Figure S5 – The top 10 canonical pathways enriched by IPA when only stage II samples were analyzed. Supplementary Figure S5A shows the canonical pathways enriched when samples from patients with stage II cancer in the right or left colon were analyzed. Supplementary Figure S5B shows the pathways enriched when samples from patients with stage II cancer in the right colon or rectum were analyzed. Supplementary Figure S5C shows the pathways enriched when samples from patients with stage II cancer in the left colon or rectum were analyzed.

A - stage II samples, right colon/left colon

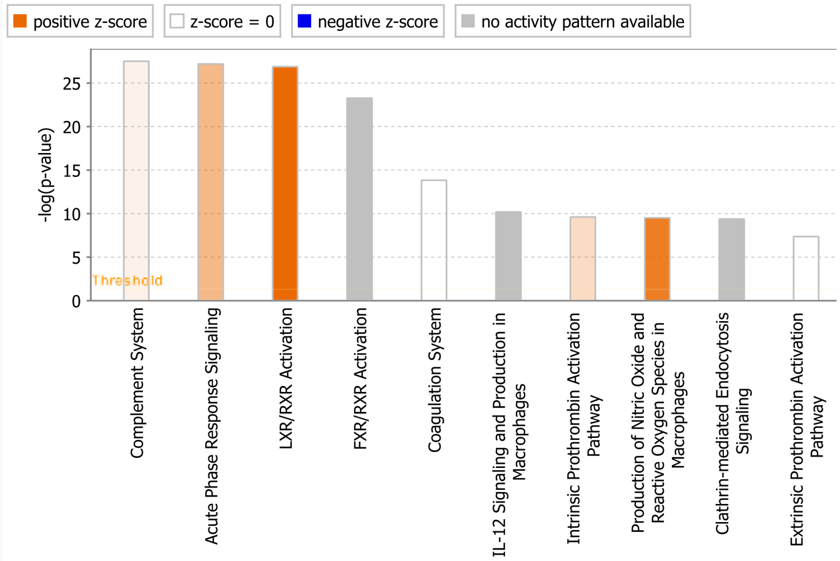

© 2000-2019 QIAGEN. All rights reserved.

B - stage II samples, right colon/rectum

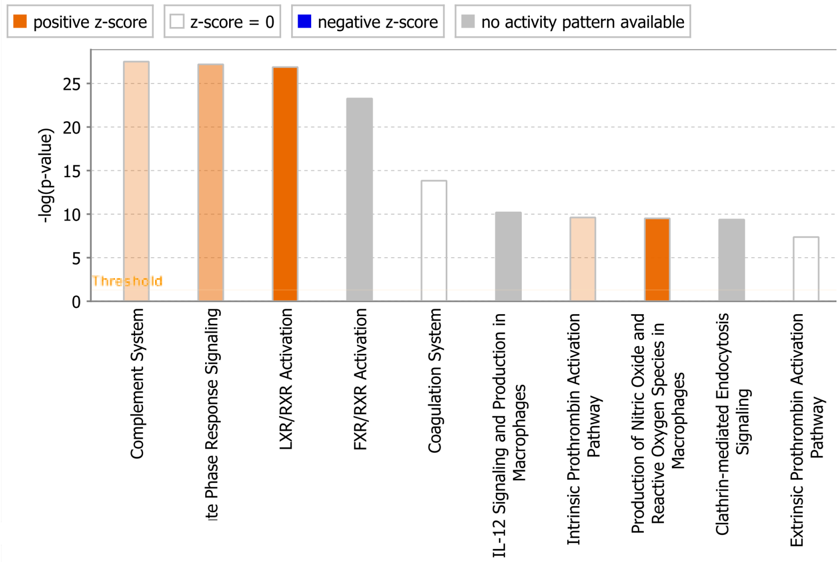

© 2000-2019 QIAGEN. All rights reserved.

C - stage II samples, left colon/rectum

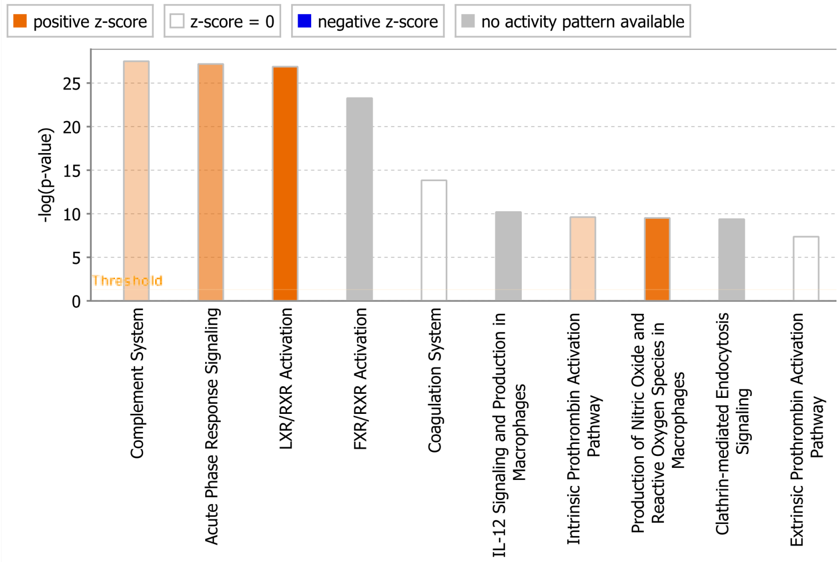

© 2000-2019 QIAGEN. All rights reserved.

Supplementary Figure S6 – The top 10 canonical pathways enriched by IPA when only stage III samples were analyzed. Supplementary Figure S6A shows the canonical pathways enriched when samples from patients with stage III cancer in the right or left colon were analyzed. Supplementary Figure S6B shows the pathways enriched when samples from patients with stage III cancer in the right colon or rectum were analyzed. Supplementary Figure S6C shows the pathways enriched when samples from patients with stage III cancer in the left colon or rectum were analyzed.

A - stage III samples, right colon/left colon

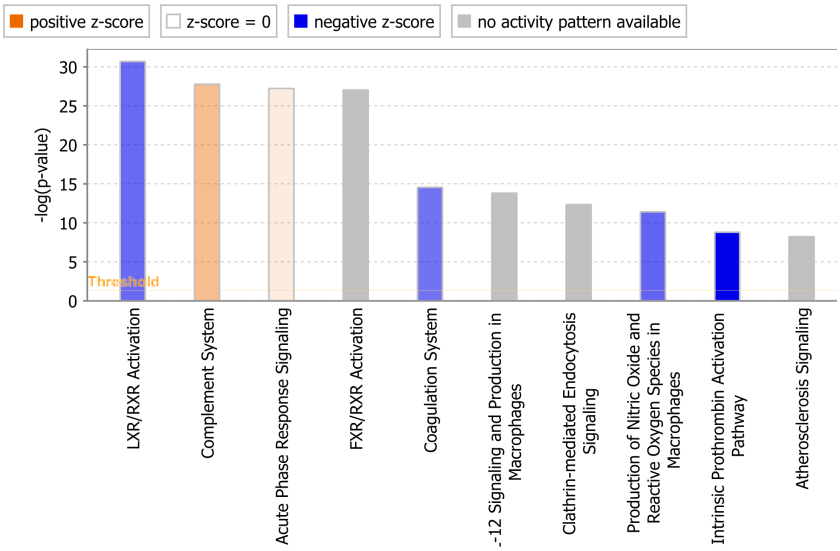

B - stage III samples, right colon/rectum

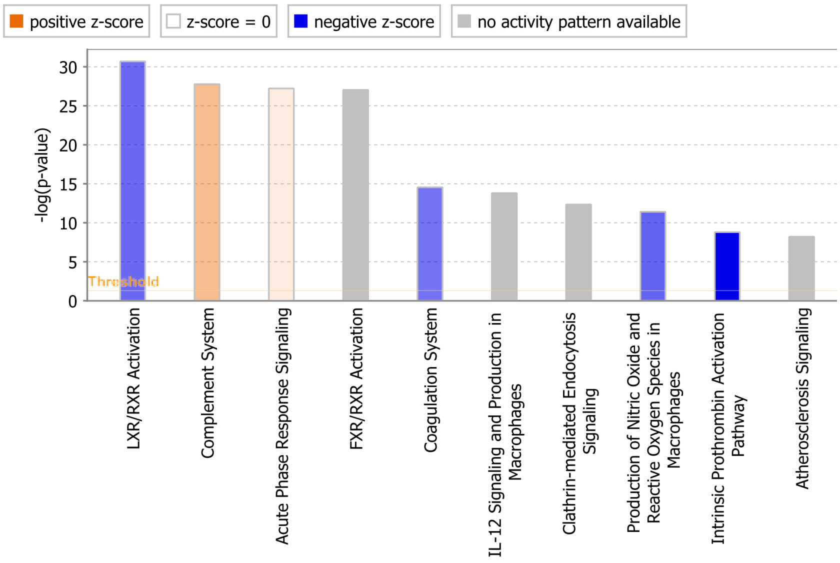

© 2000-2019 QIAGEN. All rights reserved.

C - stage III samples, left colon/rectum

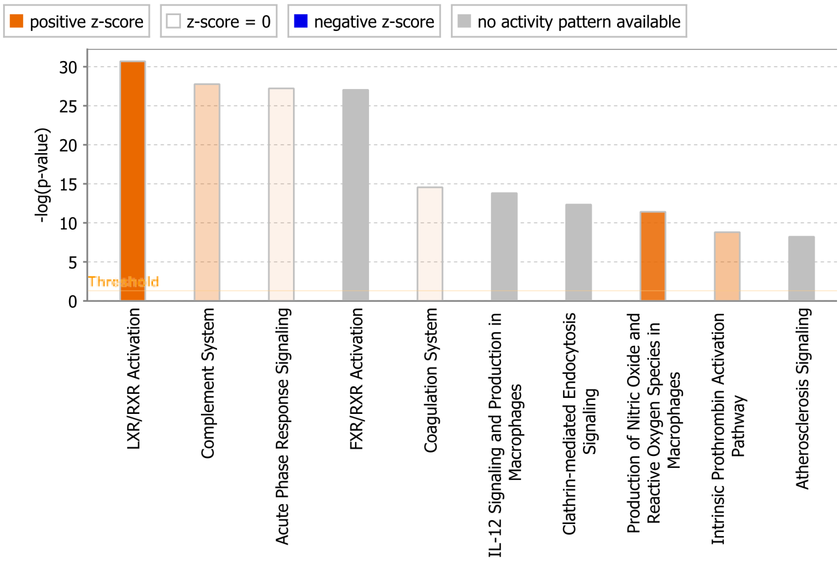

© 2000-2019 QIAGEN. All rights reserved.
